# High incidence of barotrauma in patients admitted with COVID-19 to ICU and associated mortality in rural Appalachia: An observational study

**DOI:** 10.1371/journal.pone.0282735

**Published:** 2023-03-09

**Authors:** Sunil Sharma, Varun Badami, Edward Rojas, Rahul Sangani, Kyle Chapman, Carlo Avalon, Austin King, Sijin Wen

**Affiliations:** 1 Division of Pulmonary Critical Care and Sleep Medicine, West Virginia University, Morgantown, WV, United States of America; 2 West Virginia University Critical Care and Trauma Institute, Morgantown, WV, United States of America; 3 Department of Medicine, West Virginia University, Morgantown, WV, United States of America; 4 Department of Biostatistics, School of Public Health, West Virginia University, Morgantown, WV, United States of America; Sant Anna Hospital: Clinica Sant’Anna, SWITZERLAND

## Abstract

**Objectives:**

To assess the incidence of barotrauma and its impact on mortality in COVID-19 patients admitted to ICU.

**Design:**

Single-center retrospective study of consecutive COVID-19 patients admitted to a rural tertiary-care ICU. The primary outcomes were incidence of barotrauma in COVID-19 patients and all-cause 30-day mortality. Secondary outcomes were the length of stay (LOS) in the hospital and ICU. Kaplan-Meier method and log-rank test were used in the survival data analysis.

**Setting:**

Medical ICU, West Virginia University Hospital (WVUH), USA.

**Patients:**

All adult patients were admitted to the ICU for acute hypoxic respiratory failure due to coronavirus disease 2019 between September 1, 2020, and December 31, 2020. Historical controls were ARDS patients admitted pre-COVID.

**Intervention:**

Not applicable.

**Measurements and main results:**

One hundred and sixty-five consecutive patients with COVID-19 were admitted to the ICU during the defined period, compared to 39 historical non-COVID controls. The overall incidence of barotrauma in COVID-19 patients was 37/165 (22.4%) compared to 4/39 (10.3%) in the control group. Patients with COVID-19 and barotrauma had a significantly worse survival (HR = 1.56, p = 0.047) compared to controls. In those requiring invasive mechanical ventilation, the COVID group also had significantly higher rates of barotrauma (OR 3.1, p = 0.03) and worse all-cause mortality (OR 2.21, p = 0.018). COVID-19 with barotrauma had significantly higher LOS in the ICU and the hospital.

**Conclusions:**

Our data on critically ill COVID-19 patients admitted to the ICU shows a high incidence of barotrauma and mortality compared to the controls. Additionally, we report a high incidence of barotrauma even in non-ventilated ICU patients.

## Introduction

Since December 31, 2019, when China reported a series of cases of acute respiratory failure caused by a new species of coronavirus, SARS-CoV-2, more than 50 million new cases, and almost 1,260,000 deaths have been confirmed worldwide [1]. Given the significant rise in the caseload of acute respiratory distress syndrome (ARDS) and widespread requirement of ventilatory support, the consequences of invasive ventilation and disease-specific factors are of particular importance. Of these, ventilator-induced lung injury and barotrauma have been of increasing interest.

The diagnosis of barotrauma is both clinical and radiological, with complications ranging from pneumothorax, pneumomediastinum, to subcutaneous emphysema. Determinants of barotrauma include transpulmonary pressures, tidal volume and presence and degree of dynamic hyperinflation. Lung protective strategies are essential to prevent barotrauma [2, 3]. Since the institution of lung-protective ventilatory strategies outlined in the ARDSnet protocols, the prevalence of barotrauma in patients with ARDS is reported at 4–11% [4–6].

Interestingly, patients with acute respiratory failure due to COVID-19 have been observed to have higher rates of barotrauma [7–10]. A recent study showed an increased incidence of barotrauma in patients with COVID-19 related ARDS, as well as associations with increased mortality and hospital length of stay (LOS) [11]. However, these studies only included patients on invasive mechanical ventilation. Additionally, there have been reports of barotrauma in patients with COVID-19 requiring high flow nasal cannula or non-invasive ventilatory support [12]. The goal of the study was to determine if patients with respiratory failure due to COVID-19 admitted to ICU had an a) increased incidence of barotrauma in both ventilated and non-ventilated patients, b) was barotrauma associated with worse outcome (mortality, LOS in ICU and LOS in the hospital).

## Methods

### Design and protocol

We conducted a single center, retrospective study with historical controls, of patients with acute hypoxic respiratory failure due to COVID-19 pneumonia who were admitted to the XXX Intensive Care Unit (XXX MICU) between September 30, 2020 to December 31, 2020. Institutional review board approval was obtained from WVU IRB (IRB # 2101205408). The electronic medical record (EMR) and imaging studies (chest x-ray and computed tomography scans) were utilized to identify all patients admitted to the MICU with acute hypoxic respiratory failure and a confirmed SARS-CoV-2 polymerase chain reaction (PCR). The primary outcome of the study was rate of barotrauma (pneumothorax, pneumomediastinum, and/or subcutaneous emphysema) and 30-day mortality in those patients with COVID-19 compared to controls. Secondary outcomes of the study included requirement of mechanical ventilation, ICU and hospital LOS. It is important to note that as per the ICU mechanical ventilation protocol all patients were placed on low tidal volume ventilation (4–6 ml/kg/ body weight) and PEEP was set to keep the driving pressures between 14–18 cm of H2O.

### Population and data collection

Inclusion criteria were all adult (≥ 18 years old) patients with acute hypoxic respiratory failure admitted to the MICU. Exclusion criteria were defined as age <18 years, active pregnancy, incarceration, and incidental PCR positivity without hypoxia. Barotrauma was identified by chart review and confirmed by imaging (CXR or CT scan of the chest). Barotrauma detected post-procedure was excluded from the study.

We defined historical controls as adult patients hospitalized with a primary diagnosis of ARDS (as defined by Berlin criteria) in 2018 and 2019 prior to the onset of the COVID-19 pandemic, admitted to the MICU.

We collected baseline demographic data, comorbid conditions, measures of illness including Sequential Organ Failure Assessment (SOFA) score, and measures of oxygenation and hypoxia including PaO_2_/FiO_2_ (P/F) ratio. Support requirements for all patients including use of non-invasive positive pressure ventilation (NIPPV), high flow nasal cannula (HFNC), invasive mechanical ventilation (IMV) and extracorporeal membranous oxygenation (ECMO) were also collected.

### Statistical analysis

Statistical analysis was conducted using SAS 9.1 (SAS institute, Cary, NC) and R (version 3.63, R Foundation, Vienna, Austria). Descriptive analysis was performed and reported as means with standard deviation and medians for continuous variables, and proportions for categorical variables. Continuous variables were compared between groups using the Wilcoxon’s rank test, while Chi-square test was used for categorical variables. Log-rank analysis and Kaplan-Meier curves were performed in the survival analysis including 30-day mortality. Multivariable logistic model was used on 30-day mortality, adjusting for the baseline data such as age, gender, smoking status and other patient characteristics. The fitted model was assessed using Akaike information criterion.

## Results

**Fig 1:**
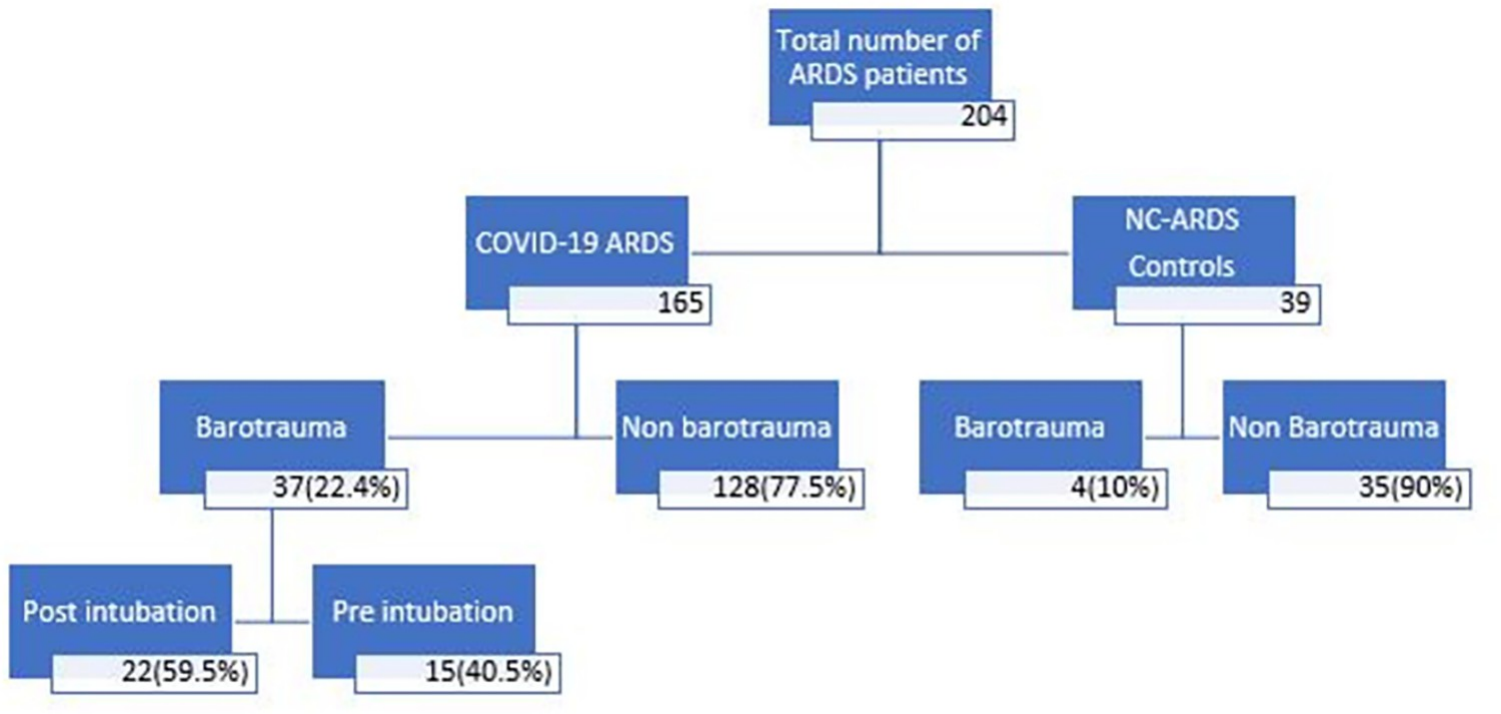
Flow chart of patients admitted for COVID -19 ARDS with control arm.

A total of 165 consecutive patients with hypoxic respiratory failure due to COVID-19 admitted to the WVUH MICU were enrolled, consisting of 113 intubated patients and 52 patients who never required intubation and mechanical ventilation. The control group constituted 39 patients admitted to the same intensive care unit in the pre-COVID era. Fig 1 describes the study approach with a focus on barotrauma.

Table 1 describes demographic and characteristic data of all COVID-19 patients (both intubated and non-intubated) compared to controls. Compared to historic controls, COVID-19 patients were older (55.4 ±12.9 vs. 66.9 ±12.4 years, respectively, p<0.01), predominantly male (41% vs. 61%, respectively, p = 0.03), and had lower body mass index (BMI, 38.9 ±12.8 vs. 33.3 ± 8.4 kg/m^2^, respectively, p = 0.01). Patients with COVID-19 had increased rates of hypertension (59% vs. 83%, p<0.01) and were less likely to be current smokers (35.9% vs.4.8%, p<0.01) compared to historic controls. Approximately one-third of patients had the comorbid chronic obstructive pulmonary disease (COPD) in the COVID-19 group which was similar to the control group. Historic ARDS control patients were sicker with higher mean SOFA score (8.8 ± 3.3 vs. 6.6 ± 3.0, p<0.01), lower mean initial P/F ratio (100.1 ± 59.0 vs. 123.7 ± 92.1, p = 0.03) and lower nadir P/F ratio during the hospital stay (67.6 ± 48.5 vs. 106.1 ± 87.2, p<0.01). Reflecting this higher severity of illness, 97.4% of historic controls required endotracheal intubation compared to 68.5% of COVID-19 ARDS patients (p<0.01). Considering treatment standards during the time period of the pandemic [13], systemic corticosteroids (10.3% vs. 92.7%, p<0.01) and remdesivir (0 vs. 41.8%, p<0.01) were used more frequently in COVID-19 ARDS patients. All patients who required prone positioning (43%) also received neuromuscular blockade with cisatracurium. Additional treatment measures such as the use of inhaled nitric oxide, need for ECMO, and tracheostomy rates were not different between the groups.

**Table 1:** Demographics and baseline characteristics of patients with COVID-19 compared to controls.

Characteristics	COVID-19	Control (non-COVID)	p-value
N	165	39	
Mean Age	66.9 ± 12.4	55.4 ± 12.9	< 0.01
Female, n (%)	64 (39%)	23 (59%)	0.03
Mean BMI	33.3 ± 8.4	38.9 ± 12.8	0.01
HTN, n (%)	137 (83%)	23 (59.0%)	< 0.01
Diabetes, n (%)	88 (53%)	15 (38.5%)	0.11
COPD, n (%)	46 (28%)	12 (30.8%)	0.7
Current smoker, n (%)	8 (4.8%)	14 (35.9%)	< 0.01
Coronary artery disease, n (%)	58 (35.2%)	9 (23.1%)	0.19
SOFA	6.6 ± 3.0	8.8 ± 3.3	< 0.01
Initial P/F	123.7 ± 92.1	100.1 ± 59.0	0.03
Worst P/F	106.1 ± 87.2	67.6 ± 48.5	< 0.01
Proning, n (%)	71 (43.0%)	23 (59.0%)	0.08
Inhaled nitric oxide, n (%)	82 (49.7%)	23 (59.0%)	0.37
Steroids, n (%)	153 (92.7%)	4 (10.3%)	< 0.01
Remdesivir, n (%)	69 (41.8%)	0	< 0.01
Tracheostomy, n (%)	17 (10.3%)	8 (20.5%)	0.18
ECMO, n (%)	9 (5.5%)	4 (10.3%)	0.28
Barotrauma pre-intubation, n (%)	15 (9.1%)	0	0.28
Barotrauma, n (%)	37 (22.4%)	4 (10.3%)	0.12

BMI–body mass index. HTN–Hypertension. COPD–Chronic obstructive pulmonary disease. SOFA–sequential organ failure assessment. P/F–PaO2/FiO2 ratio. ECMO–extracorporeal membrane oxygenation.

**Fig 2:**
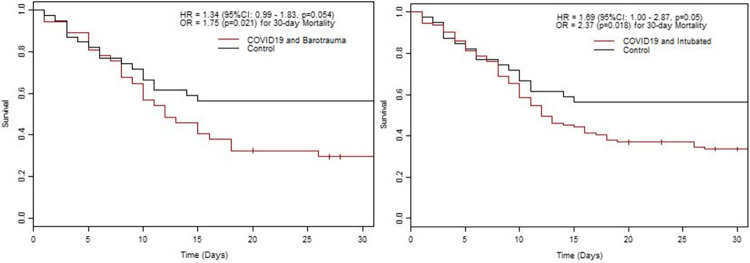
a: In the comparison to controls, patients with COVID and barotrauma had worse 30-day mortality (OR = 1.75, p = 0.021) and overall survival (HR = 1.34, p = 0.054). b: In the comparison to controls, patients with COVID and Intubated had worse 30-day mortality (OR = 2.37, p = 0.018) and overall survival (HR = 1.69, p = 0.05).

Patients with COVID-19 had a higher incidence of barotrauma than controls. A total of 37/165 (22.4%) patients with COVID-19 demonstrated barotrauma during their ICU stay, compared to 4/39 (10.3%) in the control group (OR = 2.4, p = 0.101 from Chi-square test; OR = 2.1, p = 0.033 from Mantel-Haenszel Chi-square test). Of the 37 patients with barotrauma events, 23 had pneumomediastinum, 19 had pneumothorax, 22 had subcutaneous emphysema, and 22 had a combination of events. These patients had worse overall survival (Fig 2A).

A more specific comparison of patients requiring invasive mechanical ventilation is noted in Table 2. While there was no significant difference in days of intubation, ICU LOS, hospital LOS, need for prone positioning, inhaled nitric oxide, ECMO, and tracheotomy between the groups, intubated COVID-19 patients had higher rates of barotrauma (26.5% vs. 10.5%, p = 0.03) and 30-day mortality (64.6% vs. 44.7%, p = 0.02) (Fig 2B). The intubated COVID-19 patients had worse 30-day mortality (OR = 2.21, p = 0.018) and overall poor survival (HR = 1.69, p = 0.05) compared to intubated controls (Fig 2B).

**Table 2:** Characteristics and outcomes of COVID-19 patients requiring invasive mechanical ventilation vs intubated non-COVID-19.

Characteristics	Intubated COVID-19 ARDS	Intubated Control (non-COVID)	p-value
N	113	38	
SOFA	7.3 ± 3.0	8.9 ± 3.2	< 0.01
Initial P/F	109.4 ± 77.0	100.1 ± 59.8	0.45
Worst P/F	89.7 ± 69.7	67.2 ± 49.1	0.03
Total Hospital Days	18.4 ± 18.1	18.8 ± 15.8	0.91
Total ICU Days	10.7 ± 7.0	9.3 ± 5.0	0.08
Total ventilator days, n (%)	8.4 ± 5.9	8.8 ± 6.7	0.71
Proned, n (%)	66 (58.4%)	23 (57.9%)	0.82
Inhaled nitric oxide, n (%)	65 (57.5%)	23 (57.9%)	0.75
Tracheostomy, n (%)	16 (14.2%)	8 (21.1%)	0.31
ECMO, n (%)	9 (8.0%)	4 (10.5%)	0.63
Barotrauma, n (%)	31 (26.5%)	4 (10.5%)	0.03
Mortality, n (%)	74 (64.6%)	17 (44.7%)	0.02

BMI–body mass index. HTN–Hypertension. COPD–Chronic obstructive pulmonary disease. SOFA–sequential organ failure assessment. P/F–PaO2/FiO2 ratio. ECMO–extracorporeal membrane oxygenation.

The groups of COVID-19 with and without barotrauma were explored further to determine the specific impact of barotrauma (Table 3). While observing no difference between the groups in demographics, comorbid conditions, and mean SOFA score, the group with COVID-19 and barotrauma had a lower mean initial P/F ratio (91.3 ± 45.9 vs. 137.4 ± 99.6, p<0.01) and a lower nadir P/F ratio during hospital stay (73.6 ± 36.5 vs. 115.6 ± 95.7, p<0.01). A higher proportion of the COVID-19 barotrauma patients were intubated (84% vs. 64%, p = 0.02), needed inhaled nitric oxide support (73% vs. 43%, p<0.01), and had a trend towards increased utilization of intravenous methylprednisolone (100% vs. 91%, p = 0.053). Patients with COVID-19 and barotrauma performed poorly in all reported outcome measures including days of intubation (7.8 ± 6.4 vs. 5.1 ± 6.1 days, p = 0.014), ICU LOS (11.7 ± 7.3 vs. 8.43 ± 6.51 days, p<0.01), hospital LOS (23.8 ± 26.3 vs. 14.3 ± 10.3 days, p<0.01) and 30-day mortality (70% vs. 52%, p = 0.043) as compared to COVID-19 without barotrauma.

**Table 3:** Characteristics and outcomes of patients with COVID-19 admitted to the ICU who had barotrauma compared to no barotrauma.

Characteristics	Barotrauma	No Barotrauma	p-value
N	37	128	
Mean Age	67.8 ± 11.0	66.7 ± 14.0	0.64
Female, n (%)	12 (32%)	52 (41%)	0.37
Mean BMI	32.9 ± 7.7	33.4 ± 8.6	0.78
HTN, n (%)	33 (89%)	104 (81%)	0.26
Diabetes, n (%)	17 (46%)	71 (56%)	0.29
COPD, n (%)	9 (2.4%)	37 (29%)	0.58
Current smoker, n (%)	1 (2.7%)	7 (5.5%)	0.49
Coronary artery disease, n (%)	15 (41%)	43 (34%)	0.44
SOFA score	7.0 ± 3.3	6.5 ± 2.9	0.41
Initial P/F	91.3 ± 46.0	137 ± 99.6	< 0.01
Worst P/F	73.6 ± 36.5	115.6 ± 95.7	0.01
Intubated, n (%)	31 (84%)	82 (64%)	0.02
Mean ventilator days	8.0 ± 6.4	5.1 ± 6.1	0.01
Total ICU days	11.7 ± 7.3	8.4 ± 6.5	0.01
Total Hospital days	23.8 ± 26.3	14.4 ± 10.3	< 0.01
Proned positioning, n (%)	20 (54%)	51 (40%)	0.12
Inhaled nitric oxide, n (%)	27 (73%)	55 (43%)	< 0.01
Dexamethasone, n (%)	37 (100%)	116 (91%)	0.053
ECMO, n (%)	2 (5.4%)	7 (5.5%)	0.99
Mortality, n (%)	26 (70%)	66 (52%)	0.04

We compared barotrauma and COVID-19 patients with others using a multivariable logistic regression model adjusting for potential confounding variables including COPD and current smoking status. The odds ratio for 30-day mortality in COVID-19 with barotrauma from the fitted model was 2.39 (p = 0.025).

Lastly, Table 4 (supplement) provides a detailed description of COVID-19 patients who developed barotrauma prior to invasive mechanical ventilation (n = 15, 40.5% of all barotrauma). Most patients had pneumomediastinum (67%), followed by pneumothorax (53%), and subcutaneous emphysema (47%). Only one-third of these patients were female. While one-third of patients had a history of COPD, none of them were current smokers. All patients were treated with systemic corticosteroids followed by inhaled nitric oxide (87%) for severe ARDS range initial P/F ratio (99.5 ± 21.5). 30-day mortality was significantly higher at 80% for these patients.

**Table 4:** Baseline characteristics and outcomes of patients with COVID-19 with barotrauma prior to intubation and mechanical ventilation.

Characteristics	Barotrauma prior to invasive mechanical ventilation
N	15
Mean Age	69.0 ± 11.5
Female, n (%)	4 (27%)
Mean BMI	31.4 ± 7.6
HTN, n (%)	15 (100%)
Diabetes, n (%)	8 (53%)
COPD, n (%)	5 (33%)
Current smoker, n (%)	0 (0%)
Coronary artery disease, n (%)	8 (53%)
SOFA score	5.8 ± 3.0
Initial P/F	99.5 ± 21.5
Worst P/F	81.3 ± 51.1
Pneumomediastinum, n (%)	10 (67%)
Pneumothorax, n (%)	8 (53%)
Subcutaneous emphysema, n (%)	7 (47%)
Total ICU days	10.3 ± 5.5
Total Hospital days	17.7 ± 10.1
Proned positioning, n (%)	5 (33%)
Inhaled nitric oxide, n (%)	13 (87%)
ECMO	0
Dexamethasone, n (%)	15 (100%)
Mortality, n (%)	12 (80%)

## Discussion

In this study comparing critically ill patients with COVID-19 hypoxemic respiratory failure to non-COVID ARDS controls, we show significantly increased rates of barotrauma in COVID-19 patients requiring invasive mechanical ventilatory support, significant rates of barotrauma prior to initiation of IMV, and worsened mortality.

Net rates of barotrauma were increased in patients with COVID-19. For all-comers in the COVID cohort (113 intubated patients and 52 non-intubated patients), the net rate of barotrauma was 22.4%, compared to 10.3% in the historic controls (the vast majority of whom required IMV). Furthermore, the incidence in our historical control group is consistent with published literature [14].

When comparing only the patients requiring IMV, the rates of barotrauma were 26.5% compared to 10.5%. A novel finding was that 15/37 (40.5%) patients with COVID-19 had barotrauma before intubation, while on non-invasive support such as NIPPV and/or HFNC. This is a unique and concerning finding as barotrauma with non-invasive ventilation and high flow oxygen in ARDS patients is not common [15]. We are aware of only one study which has highlighted this problem, although our incidence is double than reported by Rajdev et al. [12]. This is an important clinical finding as it may suggest an increased susceptibility of COVID-19 patients to barotrauma even in the absence of IMV. The finding of high incidence of barotrauma in patients not on invasive ventilation (NIPPV and HFNC) is important beyond ICU as during the pandemic vast number of these patients are managed on regular floor by Internists/ Hospitalists. Finally, the incidence of barotrauma in patients with COVID-19 was associated with higher 30-day mortality, increased ICU, and hospital LOS.

Our data is consistent with prior studies showing a high incidence of barotrauma in COVID-19 patients which have reported prevalence ranging from 10–40% [8, 9, 16–18]. However, most of these reports are without a frame of reference. Moreover, our estimates compared to a larger controlled study by McGuinness [9] may be conservative as we did not consider a combination of events of barotrauma in the same patient as separate events, even if they occurred after a gap of 24 hours.

The existing data on the impact of barotrauma in COVID-19 patients on mortality are conflicting. A recent study (without a control group) in an urban setting showed no difference in outcome between COVID-19 patients with barotrauma versus COVID-19 without barotrauma, although barotrauma was reported as a risk factor for overall survival [11]. In a large study of over 600 patients with the historical control group, McGuinness showed increased mortality in COVID-19 with barotrauma compared to controls, but not to COVID-19 without barotrauma. Our study shows an increase in 30-day mortality for the COVID-19 barotrauma group compared to both the historical control group as well as COVID-19 without barotrauma. The patients in this study with COVID with barotrauma had worse hypoxemia as noted in the P/F ratios, higher intubation rate, higher proportion of patients treated with inhaled nitric oxide, and longer stay in ICU when compared to those without barotrauma. Interestingly, the presence of underlying COPD did not have an appreciable impact on the occurrence of barotrauma. The diagnosis of COPD was based off chart review which may have been limited due to majority of patients being transferred from outside hospitals for tertiary care, however examination of imaging findings did not reveal any presence of cysts, bullous lesions, or any other signs of chronic concomitant lung disease. Increased LOS in both ICU and total hospital days were observed in patients with COVID-19 with barotrauma as compared to COVID-19 without barotrauma, which may add to the overall cost of care. The LOS was not increased when compared to the historic controls.

The increased incidence of barotrauma in patients with COVID-19 hypoxemic respiratory failure compared to non-COVID ARDS is suggestive of increased susceptibility to such complications. This is further underscored by the noted incidence of barotrauma in patients on non-invasive respiratory support and for patients on mechanical ventilation receiving lung-protective ventilation strategies. This may be due to pathological alterations in lung mechanics. Interestingly, prior respiratory epidemics caused by coronaviridae like severe acute respiratory syndrome (SARS) and Middle East Respiratory Syndrome (MERS) also reported a higher incidence of barotrauma which, could suggest possible coronavirus species driven alveolar injury process and associated air-leak disorders [19–21]. Previous pre-COVID studies have postulated increased viral loads in the alveoli leading to pleural porosity [22, 23]. Postmortem examinations have also noted significant upper airway injuries, airway infiltrative disease, and associated inflammatory injuries [24].

One hundred and sixty-five consecutive patients with COVID-19 with detailed documentation of SOFA scores and P/F ratio (at admission and lowest recorded) helped in risk stratification of the groups to better understand outcome data.

The limitations include a retrospective design of the study with the smaller sample size of the control group, composed of mostly ARDS from the pre-COVID era. However, no cases of ARDS other than those attributable to COVID-19 were admitted to our ICU during the study window (correlating with a local surge of the COVID-19 pandemic). The majority of our patients are transferred from smaller rural hospitals and may not have received a strictly lung-protective ventilator strategy prior to transfer [25]. Another limiting factor of the study was lack of detailed vent-desynchrony events, changes in ventilator settings, and pressure changes associated with poor compliance. This may explain possible triggering factors of the patients who experienced barotrauma after intubation. These results should not be taken as causality but only as association.

## Conclusion

In this observational study we report an increased incidence of barotrauma and mortality in patients with COVID-19 admitted to the ICU. We also report a high incidence of barotrauma in non-intubated patients (NIPPV and HFNC) which traditionally have not been associated with high risk of barotrauma. These findings are of clinical importance to services managing COVID1-9 patients outside the ICU domain. COVID-19 patients with barotrauma also demonstrated higher ICU and hospital LOS compared to COVID-19 without barotrauma. Considering various pathophysiological and clinical consequences of COVID-19 in critically ill patients, the observed significance of barotrauma should be explored further in prospective trials.

## Abbreviations

BMI: body mass index
HTN: Hypertension
COPD: Chronic obstructive pulmonary disease
SOFA: sequential organ failure assessment
P/F: PaO2/FiO2 ratio
ECMO: extracorporeal membrane oxygenation
